# Location, Location, Location: Main Chain Poly(cobaltocenium)s Outperform Side Chain Analogues

**DOI:** 10.1021/acscentsci.6c01185

**Published:** 2026-07-21

**Authors:** Edmund F. Palermo

**Affiliations:** Materials Science and Engineering, Biomedical Engineering, 8024 Rensselaer Polytechnic Institute, Troy, New York 12180, United States

## Abstract

A novel class of polymeric cobaltocenium structures were evaluated for their antimicrobial activity and toxicity toward mammalian cell lines.

## Graphical abstract

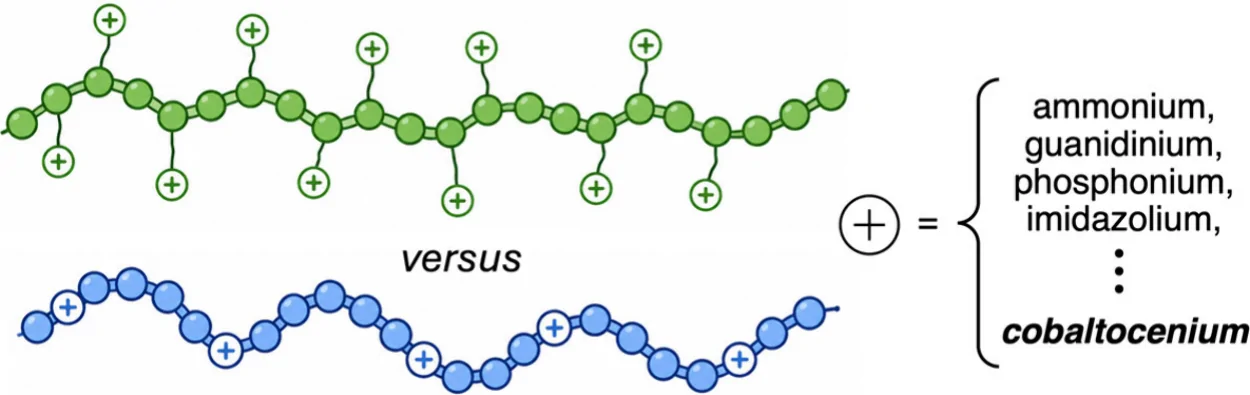

As with real estate and baseball, there are apparently three things that matter for the molecular design of cationic groups in antimicrobial polymers: location, location, location.^1,2^ In a recent issue of *ACS Central Science*, Professor Chuanbing Tang and co-workers have notably advanced the field of antimicrobial polymers through an elegant chemical approach that positions cobaltocenium groups within the main chain backbone, for the first time.^3^

The antimicrobial polymer literature dates back to at least the 1980s, when Ikeda and co-workers pioneered hydrophobic polymers bearing ammonium and phosphonium cationic groups in the side chains that kill bacteria in solution and on surfaces (Figure 1).^4^ This was, in its infancy, a very simple extension of existing benzalkonium chloride disinfectants: the benzyl group was replaced with a styrene and then polymerized by standard free radical techniques. Those materials could indeed kill bacteria at high concentrations but were also intensely toxic to human cells. Then in the 1990s, the discovery of host defense peptides (HDPs), which are also cationic and hydrophobic, showed that toxicity to humans can be solved by judicious molecular-level design.^5^ Thereafter, the field of HDP-mimetic synthetic polymers started to skyrocket in the early 2000s and has continued to grow rapidly to the present day.^6–9^ The rate of publications in this field has grown rapidly, attesting to the sustained, intense level of interest from academia and industry.

**Figure 1:**
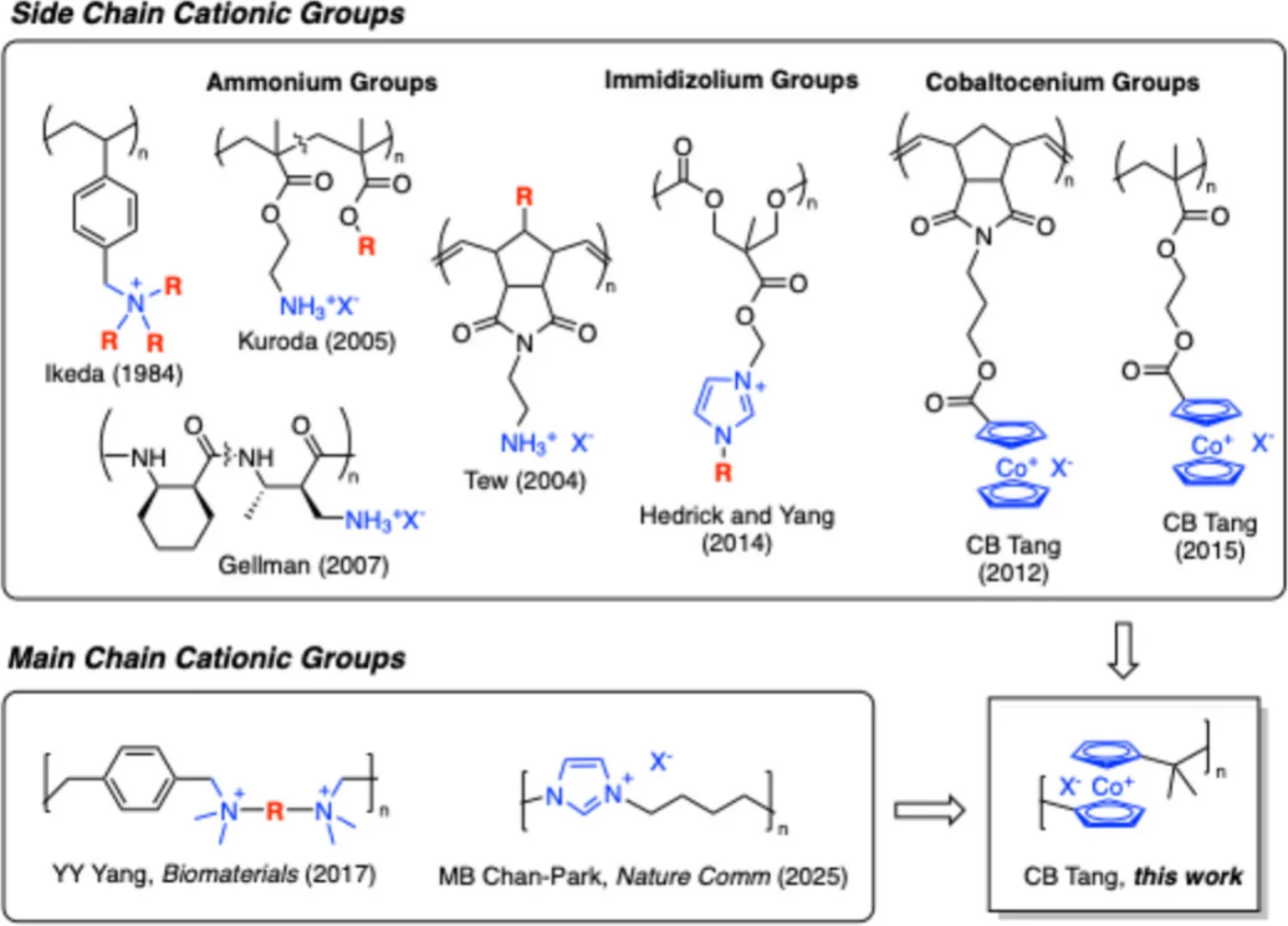
Abridged evolution of antimicrobial polycation structures, focused on the location of cationic charges either as pendant side chains or enchained in the backbone of the polymer. Four decades after the initial discovery that polycations kill bacteria, main chain cobaltoceniums with unparalleled chemical simplicity have emerged as leading candidates.

As with real estate and baseball, there are apparently three things that matter for the molecular design of cationic groups in antimicrobial polymers: location, location, location.

Myriad cationic group structures have been implemented, in conjunction with an endless variety of polymer structures and architectures, synthesized by a vast array of laboratories worldwide. By far, ammonium and phosphonium groups remain the undisputed, dominant molecular design choice for endowing cationic charges in such antimicrobial polymers. Among the vast literature on this topic, cationic groups serve an essential functional purpose. They are typically described in terms of electrostatic attraction to the negatively charged components of the bacterial cell membrane structure, a key first step in their interaction with the target microbe. Classically, these macromolecular amphiphiles are thought to disrupt the integrity of the bacterial cell membrane, leading to loss of transmembrane potential, and concomitant cell death. Other possible mechanisms may include membrane translocation and subsequent complexation with negatively charged nucleic acid polymers. Very interestingly, as Dr. Mark Chan-Park and co-workers recently unveiled, main chain poly­(imidazolium)­s with an acidic CH proton on the C2 position can potentially deprotonate to generate hydrophobic N-heterocyclic carbenes *in vivo*, leading to highly efficient membrane translocation and subsequent attack on DNA intracellularly.^10^

While the vast majority of studies have invoked ammonium or phosphonium groups as the cationic species, Tang and co-workers have uniquely produced polymer structures bearing metallocene groups as the source of cationic charge

While the vast majority of studies have invoked ammonium or phosphonium groups as the cationic species, Tang and co-workers have uniquely produced polymer structures bearing metallocene groups as the source of cationic charge.^11^ Particularly, they chose cationic cobaltocenium complexes for their excellent stability in accordance with the classical 18-electron rule. Other potential cationic metallocenes, in contrast, would likely suffer from prohibitive toxicity to human cells and/or poor chemical stability *in vivo*. The advantages of this creative, paradigm-shifting approach include the ability to conjugate with traditional antibiotic drugs to resensitize microbes against previously obsolete drugs, improved circulation and stability *in vivo*, and the inherent decoupling of amphiphilicity and total number of charges.

In this new study, the Tang group has leveraged a key synthetic breakthrough to show that changing the location of the cobaltocenium groups, from pendant dangling side chains to direct incorporation into the main chain of the polymer, has a profound effect on bioactivity: whereas the main chain location endows excellent activity in the low μg/mL concentration range, side chain analogs showed weak or no antibacterial activity.

In this new study, the Tang group has leveraged a key synthetic breakthrough to show that changing the location of the cobaltocenium groups, from pendant dangling side chains to direct incorporation into the main chain of the polymer, has a profound effect on bioactivity: whereas the main chain location endows excellent activity in the low μg/mL concentration range, side chain analogs showed weak or no antibacterial activity**.** The obtained main chain poly­(cobaltocenium)­s are deceptively simple in structure: the repeating unit contains nothing more than the cobaltocenium alternating with a geminal dimethyl group. These novel polymeric materials showed rapid, broad-spectrum activity via the classic membrane disruption mechanism, did not induce antibiotic resistance even after many subinhibitory passages, and showed substantial activity against biofilms. Although future studies on cytotoxicity and proof of efficacy *in vivo* in animal models of bacterial infection are still forthcoming, the novelty of structures and excellent bioactivity unveiled in this study, combined with the established advantages of cobaltoceniums in general, have breathed new life into this well-established field of research.

However, the “Holy Grail” of this field remains elusive: to date, the systemic administration of HDP-mimetic polymers for the treatment of bacterial infections has not yet been proven safe and effective in humans.

More than four decades since the discovery that cationic, amphiphilic polymers are intrinsically antibacterial, there are many success stories that inspire pride and continued techno-optimism. Commercialized plastic coatings on the market boast the ability to disinfect themselves on contact. In the realm of biomedical engineering research, examples abound of potential use as topical formulations in the context of wound healing, as coatings on frequently touched surfaces in hospitals, and as inherently infection-resistant coatings on in-dwelling materials including catheters, dental materials, and sutures. However, the “Holy Grail” of this field remains elusive: to date, the systemic administration of HDP-mimetic polymers for the treatment of bacterial infections has not yet been proven safe and effective in humans. The extent to which the author remains hopeful that the community will someday accomplish that long sought-after objective is bolstered by many recent advances that bring real novelty to a mature field of scientific investigations. This latest work in *ACS Central Science* is indeed a powerful testament to the value of persistence and creativity on the endless frontier.
